# Functional Diversity and Invasive Species Influence Soil Fertility in Experimental Grasslands

**DOI:** 10.3390/plants9010053

**Published:** 2020-01-01

**Authors:** Leonardo H. Teixeira, Florencia A. Yannelli, Gislene Ganade, Johannes Kollmann

**Affiliations:** 1 Restoration Ecology, Department of Ecology and Ecosystem Management, Technical University of Munich, Emil-Ramann-Str. 6, 85350 Freising, Germany; florenciayannelli@gmail.com (F.A.Y.); jkollmann@wzw.tum.de (J.K.); 2 Department of Ecology, Center for Biosciences, Federal University of Rio Grande do Norte, Senador Salgado Filho avenue, Natal/RN CEP 59078-900, Brazil; gganade@gmail.com; 3 Jeschke group-Ecological Novelty, Department of Biology, Chemistry, Pharmacy, Institute of Biology, Free University of Berlin, Königin-Luise-Str. 1-3, 14195 Berlin, Germany; 4 Norwegian Institute of Bioeconomy Research (NIBIO), P.O. Box 115, 1431 Ås, Norway

**Keywords:** biotic resistance, competition, complementarity, *Solidago gigantea*

## Abstract

Ecosystem properties can be positively affected by plant functional diversity and compromised by invasive alien plants. We performed a community assembly study in mesocosms manipulating different functional diversity levels for native grassland plants (communities composed by 1, 2 or 3 functional groups) to test if functional dispersion could constrain the impacts of an invasive alien plant (*Solidago gigantea*) on soil fertility and plant community biomass via complementarity. Response variables were soil nutrients, soil water nutrients and aboveground biomass. We applied linear mixed-effects models to assess the effects of functional diversity and *S. gigantea* on plant biomass, soil and soil water nutrients. A structural equation model was used to evaluate if functional diversity and invasive plants affect soil fertility directly or indirectly via plant biomass and soil pH. Invaded communities had greater total biomass but less native plant biomass than uninvaded ones. While functional diversity increased nutrient availability in the soil solution of uninvaded communities, invasive plants reduced nutrient concentration in invaded soils. Functional diversity indirectly affected soil water but not soil nutrients via plant biomass, whereas the invader reduced native plant biomass and disrupted the effects of diversity on nutrients. Moreover, invasive plants reduced soil pH and compromised phosphate uptake by plants, which can contribute to higher phosphate availability and its possible accumulation in invaded soils. We found little evidence for functional diversity to constrain invasion impacts on nutrients and plant biomass. Restoration of such systems should consider other plant community features than plant trait diversity to reduce establishment of invasive plants.

## 1. Introduction

The functional diversity of a plant community can be seen as a good predictor of ecosystem functioning [1,2]. Increased functional diversity, measured by plant traits, positively affects nutrient cycling and storage [3,4], increases soil fertility [4], and enhances plant productivity [5]. The complementarity resulting from plant functional diversity is an important driver of diversity-productivity relationships, and this can lead to a more even use of limiting resources [1,6,7]. Therefore, by controlling above and belowground biomass production and, consequently, nutrient retention and acquisition, plant traits can influence ecosystem productivity even when facing perturbations [8,9,10]. In the past decade, one of the most common perturbation sources is the invasion of degraded, restored or native systems by exotic plants [11]. In most cases, the aforementioned ecosystem properties are frequently impacted by invasive plant species [12], in both short and long term [13].

On one hand, functional diversity has been linked to the reduction of growth and spread of invasive species [14] and also to the increase of biotic resistance of plant communities via resource depletion [11,15]. On the other hand, invasive species can reduce native plant biomass by competition [16,17], decreasing nutrient uptake of native plants [18] and altering nutrient cycling, e.g., due to higher phosphorus turnover [19,20]. Such impacts on soil nutrient balance potentially lead to positive feedbacks favoring the persistence of invasive species, while hampering the recovery of disturbed native communities [21]. 

Ultimately, the successful invasion of plant communities could be defined by the capacity of some invasives to produce more biomass than native species [22]. To achieve this goal, highly successful invaders like *Solidago gigantea* can change soil nutritional conditions, e.g., by altering pH values and nutrient availability in invaded soils [19], thus, limiting native plant germination and growth while increasing its own biomass production [23]. However, functional diversity of native plants can buffer the impacts of invasive species on ecosystem functioning [14,15]. For instance, higher functional diversity has been linked to a reduction in soil acidity leading to a higher consumption of nutrients by native species, reducing nutrient availability and exerting a strong control on soil fertility [24]. Therefore, increased biotic resistance would be expected due to marked competition and depletion of limiting resources that would otherwise be used by the invader [15]. 

Although life strategies and other characteristics of invasive plants (e.g., larger size, higher growth rate and resource-use acquisition) can influence their impact on invaded systems [12], it has been pointed out that the effects of invaders on ecosystem functioning might not only depend on its characteristics but rather on site conditions [23]. For instance, changes in land use have altered species composition of mesic European grasslands in many regions, leading to alien plant invasion [25]. Furthermore, areas allocated for grassland restoration are often prone to the invasion of weeds and other unwanted invasive species. Therefore, experiments with model grasslands are needed for improving restoration methods and for designing plant communities that would be most efficient in restoring invaded grasslands and resisting to their impacts [26]. Such experiments should test functional diversity of seed mixtures and identify species that could reduce plant invasions [27].

**Figure 1:**
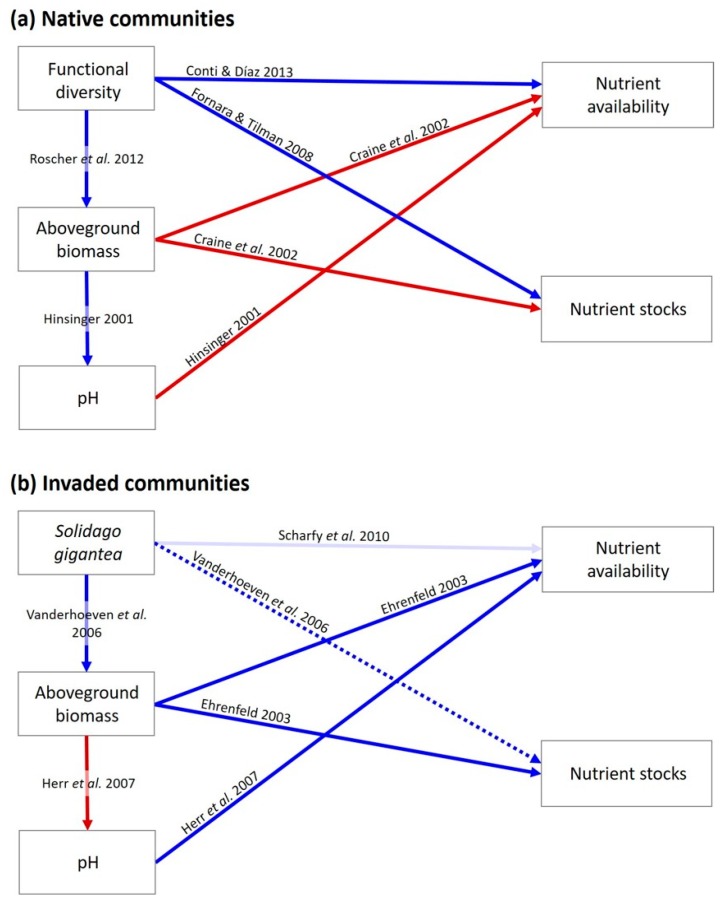
Hypothesised causal relationships of functional diversity of native plants, the invasive alien *Solidago gigantea*, plant aboveground biomass and soil pH with soil nutrient availability and stocks for (**a**) uninvaded [3,4,5,9,24] and (**b**) invaded communities [19,26,29,30]. Arrows represent unidirectional relationships among variables, blue arrows show positive and red arrows show negative relationships. Solid lines indicate significant paths (*p* ≤ 0.05), and dashed lines indicate marginally significant paths (*p* ≤ 0.10), while transparent arrows represent non-significant paths (*p* > 0.10).

Focusing on the impacts of an invasive plant species on plant community biomass and soil nutrients when invading grasslands communities with different levels of diversity, we did a community assembly experiment in mesocosms by manipulating native plants functional diversity (represented by the number of functional groups composing the seed mixtures used for making up the communities) and invasion by *S. gigantea*. For these communities, we used the functional dispersion index (Fdis; [28]) as a measure of functional diversity to test if biotic resistance will be increased by complementarity effects, thus, reducing the impacts of invasive plants on productivity and soil fertility. Functional dispersion indicates the degree in which plant species occupy the multidimensional trait space, representing a community composed by functionally dissimilar species and, therefore, having higher ecological niche occupancy [6,28]. Therefore, we hypothesized that: (i) Functional dispersion will increase native plant biomass due to complementarity, thus increasing biotic resistance of grassland communities because of higher niche occupancy. (ii) Functional diversity will bear a strong control over soil fertility in uninvaded communities due to complementarily using resources and positively affecting biomass of native plants, thus reducing nutrient availability and accumulation in the soil. (iii) Invasive alien plants will increase productivity and soil fertility in invaded communities, while reducing native plant biomass by competition. (iv) Invaded communities will lose the control of native functional diversity on soil fertility due to the negative effects of the invader on native plants’ biomass. Furthermore, by means of structural equation models (SEM) we investigated if the effects of plant functional diversity and *S. gigantea* would be either direct on soil and soil water nutrients or indirectly mediated by plant aboveground biomass and soil pH. Finally, we evaluated whether or not the direct and indirect effects of functional diversity on nutrients are negatively affected by *S. gigantea*. In other words, we investigated if the control exerted by plant diversity would remain the same or would be disrupted when comparing native and invaded communities (Figure 1).

## 2. Results

### 2.1. Effects on Plant Emergence and Aboveground Biomass

**Figure 2:**
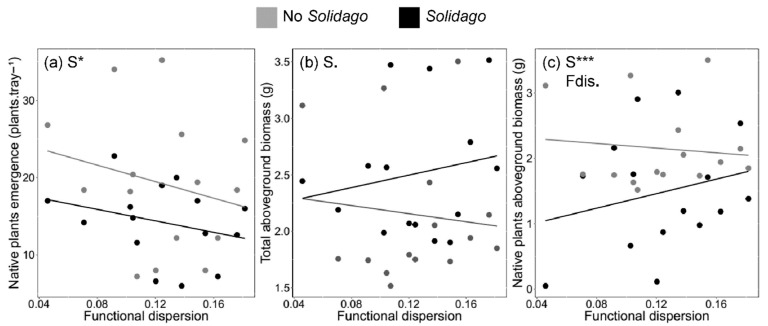
Effects of functional diversity, represented by the functional dispersion (Fdis) index and *Solidago gigantea* (S) on plants emergence and aboveground biomass; (**a**) shows native plant emergence according to functional dispersion and the presence of the invasive alien plant, while (**b**) shows native plant biomass in relation to functional dispersion and the presence of the invader; (**c**) shows plant communities total aboveground biomass varying according to the functional dispersion and *S. gigantea*. Aboveground biomass was collected at the end of the experiment (16 weeks), while emergence was evaluated during the second week of the experimental period for each one of the communities, invaded or not. For the dependent variables, chi-squared values are presented in the text (*** *p* ≤ 0.001; * *p* ≤ 0.05; *p* ≤ 0.10).

Overall, native plant emergence was 26% higher when comparing uninvaded communities to invaded ones (19.3 ± 3.97 [±SE] and 14.3 ± 2.20 emerged plants per tray, respectively). Emergence of native plants was negatively affected by *S. gigantea*, but functional dispersion (Fdis) did not influence native plant emergence (*χ*^2^ = 46.9, *df* = 7, *p* ≤ 0.001 for *Solidago* effects; *χ*^2^ = 0.004, *df* = 7, *p* > 0.10 for Fdis effects; Figure 2a). Although no significant effects, we observed a trend for higher functional dispersion to reduce native plants (Figure 2a) and *S. gigantea* emergence (Supplementary File 1; Figure S1).

At the end of the experimental period, invaded communities had on average 26% more plant biomass than uninvaded ones (for invaded communities: 14.5 ± 4.3 g of dry plant mass; for uninvaded communities: 10.8 ± 4.0 g of dry plant mass). In fact, presence of *S. gigantea* marginally increased total aboveground biomass of invaded plant communities, while functional diversity dispersion did not (*χ*^2^ = 3.6, *df* = 7, *p* = 0.056 for *Solidago* effects; *χ*^2^ = 1.4, *df* = 7, *p* > 0.10 for Fdis effects; Figure 2b). On the other hand, native plant biomass was on average 40% higher in uninvaded communities in comparison to the biomass values found for invaded communities (10.8 ± 4.0 g and 6.4 ± 2.7 g, respectively). We found aboveground biomass of native plants to be marginally positively affected by increased functional dispersion (*χ*^2^ = 3.6, *df* = 7, *p* = 0.057; Figure 2c). Still, the influence of functional dispersion on native plant biomass was only detected when plant communities were invaded by *S. gigantea*. Additionally, native plant biomass was significantly negatively affected by the presence of *S. gigantea* (*χ*^2^ = 11.2, *df* = 7, *p* ≤ 0.001). In fact, the presence of the invader reduced aboveground biomass of native plants by 68% across all treatments (Figure 2c), while no effect of functional dispersion on *S. gigantea* biomass was observed (*F* = 0.58, *df* = 8, *p* > 0.10, Supplementary File 1; Figure S1). Finally, total emergence had no significant effects on total biomass (estimate = 0.001, *t* = 0.15), native biomass (estimate = 0.01, *t* = 1.4) and *S. gigantea* biomass models (*F* = 0.01, *df* = 8, *p* > 0.10).

### 2.2. Direct and Indirect Effects of Functional Diversity and S. gigantea on Nutrients

**Figure 3:**
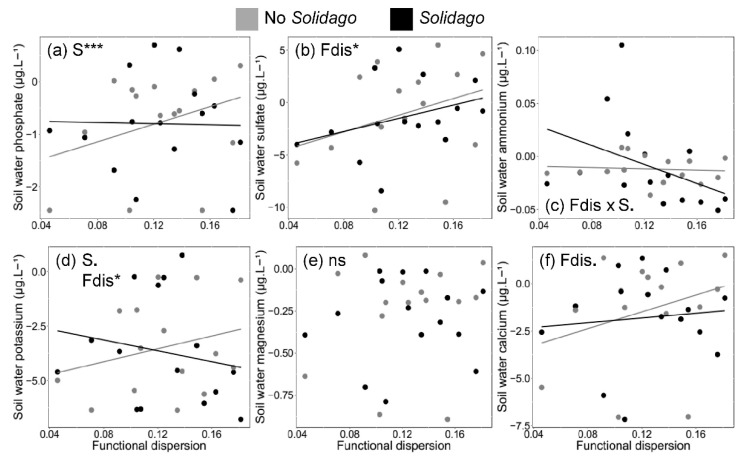
Effects of functional dispersion (Fdis) and *Solidago gigantea* (S) on soil water macronutrients measured from the grassland communities. Figures show six macronutrients that are important for plant growth and are directly controlled by plants: (**a**) phosphate; (**b**) sulfate; (**c**) ammonium; (**d**) potassium; (**e**), magnesium; and (**f**), calcium. For the dependent variables, chi-squared values are presented in the text (*** *p* ≤ 0.001; * *p* ≤ 0.05; *p* ≤ 0.10; ns *p* > 0.10).

Functional diversity (measured by functional dispersion) increased nutrient concentration in soil water of uninvaded communities by 14% on average in comparison to invaded communities when considering all nutrient forms (Figure 3). However, linear mixed-effects models revealed that from the six macronutrients measured, functional dispersion significantly increased only in two macronutrients. Sulfate and potassium concentrations were significantly increased under increasing functional dispersion of native plants (for sulfate: *χ*^2^ = 5.4, *df* = 7, *p* ≤ 0.05; Figure 3b; for potassium: *χ*^2^ = 4.0, *df* = 7, *p* ≤ 0.05; Figure 3d), while the concentration of calcium only marginally significantly increased with functional dispersion (*χ*^2^ = 3.2, *df* = 7, *p* = 0.07; Figure 3f). Soil water ammonium was, in turn, marginally significantly reduced due to the interaction between functional dispersion and *S. gigantea* in invaded communities under increasing functional dispersion (*χ*^2^ = 3.3, *df* = 8, *p* = 0.07; Figure 3c). Moreover, soil water phosphate was significantly reduced due to the presence of *S. gigantea* when comparing invaded and uninvaded communities with high values of functional dispersion (*χ*^2^ = 15.4, *df* = 7, *p* ≤ 0.001; Figure 3a). Finally, *S. gigantea* marginally significantly reduced potassium concentrations in the soil water (*χ*^2^ = 3.1, *df* = 7, *p* = 0.08; Figure 3d), while soil water magnesium was not affected by functional dispersion (*χ*^2^ = 0.05, *df* = 7, *p* > 0.10) nor by *S. gigantea* (*χ*^2^ = 0.001, *df* = 7, *p* > 0.10; Figure 3e).

**Figure 4:**
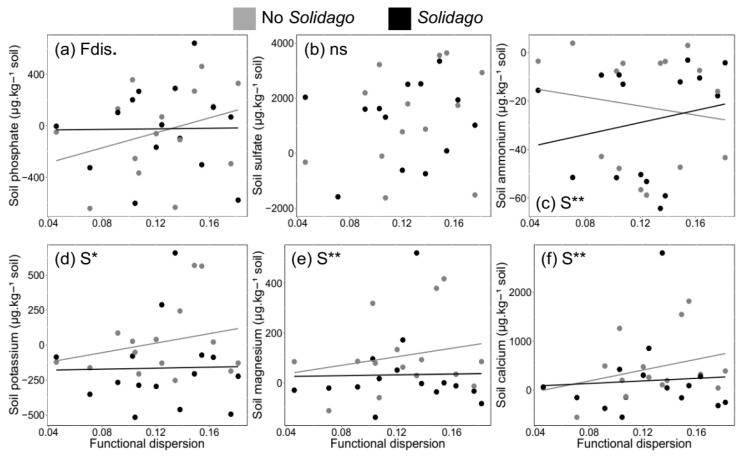
Effects of functional dispersion (Fdis) and *Solidago gigantea* (S) on the macronutrients measured in the soil of the grassland communities. Figures show six macronutrients that are important for plant growth and are directly controlled by plants: (**a**), phosphate; (**b**), sulfate; (**c**), ammonium; (**d**), potassium; (**e**), magnesium; and (**f**), calcium. For the dependent variables, chi-squared values are presented in the text (** *p* ≤ 0.01; * *p* ≤ 0.05; *p* ≤ 0.10; ns *p* > 0.10).

Overall, invaded communities had 54% less nutrients in the soil than uninvaded ones when considering all nutrient forms across the functional dispersion values. Functional diversity (measured by functional dispersion) did not control soil nutrients in the mesocosms (Figure 4), except for soil phosphate in uninvaded communities that was marginally increased under increasing functional dispersion (*χ*^2^ = 3.5, *df* = 7, *p* = 0.06; Figure 4a). Differently, the presence of *S. gigantea* reduced concentrations in the soil for four out of six macronutrients measured (Figure 4c–f). While soil ammonium concentration increased in invaded communities with high values of functional dispersion (*χ*^2^ = 7.0, *df* = 7, *p* ≤ 0.01; Figure 4c), potassium (*χ*^2^ = 4.6, *df* = 7, *p* ≤ 0.05; Figure 4d), magnesium (*χ*^2^ = 10.4, *df* = 7, *p* ≤ 0.01; Figure 4e) and calcium (*χ*^2^ = 7.5, *df* = 7, *p* ≤ 0.01; Figure 4f) concentrations were significantly reduced in the soil of invaded communities independently of functional dispersion. Finally, soil sulfate concentration was not affected by functional dispersion (*χ*^2^ = 2.0, *df* = 7, *p* > 0.10) nor by *S. gigantea* (*χ*^2^ = 0.83, *df* = 7, *p* > 0.10; Figure 4b).

**Figure 5:**
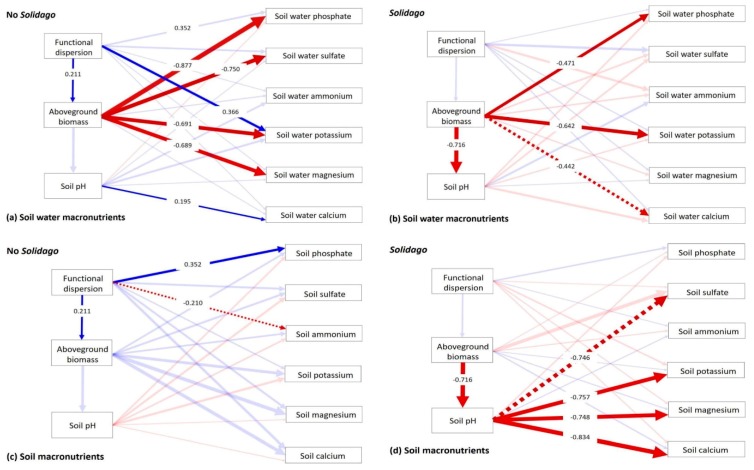
Structural equation model (SEM) for the effects of functional dispersion and *Solidago gigantea* on the macronutrients in the soil water fraction (**a**,**b**) and in the soil of the grassland communities (**c**,**d**). Unidirectional relationships among variables are represented by the arrows, and path coefficients indicate the strength of the correlations [31]. Arrow thickness reflects scaled values of coefficients and solid arrows represent significant effects (*p* ≤ 0.05). Blue arrows represent positive and red arrows negative effects. Dotted arrows show marginally significant (*p* ≤ 0.1) and transparent arrows non-significant effects (*p* > 0.10).

The structural equation models indicated that functional dispersion of native communities indirectly controlled soil water nutrients via plant biomass (Figure 5a,b). In fact, functional dispersion positively controlled aboveground biomass in uninvaded communities (estimate = 0.211, *p* ≤ 0.05; Figure 5a,c). In both, invaded and uninvaded communities, functional dispersion did not directly affect soil water nutrients except for positive effects on soil water potassium in uninvaded communities (estimate = 0.366, *p* ≤ 0.05; Figure 5a).

For native communities, aboveground biomass negatively affected soil water phosphate (estimate = −0.877, *p* ≤ 0.001), sulfate (estimate = −0.750, *p* ≤ 0.01), potassium (estimate = −0.691, *p* ≤ 0.05) and magnesium (estimate = −0.689, *p* ≤ 0.05), while soil pH positively affected calcium availability (estimate = 0.195, *p* ≤ 0.05; Figure 5a). For invaded communities, presence of *S. gigantea* disrupted the effect of functional dispersion on aboveground biomass and created a negative effect of biomass on soil pH (estimate = −0.716, *p* ≤ 0.01; Figure 5b,d). As a consequence, effects on soil water macronutrients were altered (Figure 5b). By the influence of *S. gigantea*, biomass control of soil water phosphate was reduced nearly by half (estimate = −0.471, *p* ≤ 0.05), while biomass control of soil water potassium remained similar (estimate = −0.642, *p* ≤ 0.01). However, *S. gigantea* disrupted direct effects of functional dispersion on soil water potassium and biomass control of soil water sulfate and magnesium. Moreover, in invaded communities, positive effects of soil pH on soil water calcium were lost, and a marginal negative effect of biomass occurred in turn (estimate = −0.442, *p* = 0.06; Figure 5b).

Most of the nutrients in the soil of native communities were not affected by functional dispersion, with the exception for a positive effect on soil phosphate (estimate = 0.352, *p* ≤ 0.05) and a marginal negative effect on soil ammonium (estimate = −0.210, *p* = 0.06; Figure 5c). For invaded communities, negative influence of biomass on soil pH induced by *S. gigantea* resulted in an indirect effect of aboveground biomass on soil nutrients via soil pH (Figure 5d). In fact, soil pH marginally negatively affected soil sulfate (estimate = −0.746, *p* = 0.06) and significantly negatively affected potassium (estimate = −0.757, *p* ≤ 0.05), magnesium (estimate = −0.748, *p* ≤ 0.05) and calcium (estimate = −0.834, *p* ≤ 0.01).

## 3. Discussion

### 3.1. Effects on Plant Emergence and Aboveground Biomass

Our findings did not support the idea that increased functional diversity of native plants (measured by functional dispersion) could constrain invasion impacts by enhancing competitive interaction with invasive plants or due to an exhaustive use of limiting resources resulting from greater native plant biomass [14,15]. While the emergence of native plants was compromised by the presence of invasive plants, we only found a weak trend for emergence of *S. gigantea* to reduce in communities with higher functional dispersion (Supplementary File 1; Figure S1). In fact, previous results found invasive plants emerging earlier than native species, thus altering conditions in the invaded soils and impacting native plant emergence [32]. Such pattern can create a window of opportunity for invasive species to outcompete native species during early stages of invasion [11]. Though our findings indicate that functional dispersion did not increase biotic resistance to constrain *S. gigantea* invasion impacts, there is ample evidence that justifies the inclusion of functional diversity aspects in grassland restoration [15,33].

We observed that the presence of *S. gigantea* marginally increased total aboveground biomass of invaded plant communities, while functional dispersion did not. Such results indicate that *S. gigantea* can initially increase productivity of invaded communities. However, if considered in the long term, effects of this invasive species on native plants emergence rates and biomass production can reduce diversity of invaded communities by competitive exclusion of native species, thus deteriorating biotic resistance of such communities and, possibly, creating opportunities for further invasions [18,19,23]. Furthermore, a recent study found an invasive plant to strongly affect the functional composition of native communities during early stages of development [34]. 

In our experiment, presence of *S. gigantea* negatively affected native plant biomass, independently of the community functional diversity, even if there was a marginal increase on the total aboveground biomass of invaded communities. Thus, our study points to a suppression effect of *S. gigantea* due to more biomass and an increased nutrient turnover [18,19,21,23]. Competitive effects of invasive plants on native species can be stronger than vice versa [17], which was confirmed in our study since *S. gigantea* negatively affected native plants, but the opposite was not observed. Although native and invasive alien plants were sown with different seed densities (3 g m^−2^ and 1 g m^−2^, respectively), *S. gigantea* seeds are much smaller than the seeds of the native species used here. Thus, the actual number of seeds per area was probably higher for *S. gigantea* perhaps leading to higher potential emergence of invasive plants. Finally, our results agree with other studies showing that *S. gigantea* produce more biomass than native species in invaded sites [29,35]. 

### 3.2. Direct and Indirect Effects of Functional Diversity and S. gigantea on Soil Fertility

Previous studies showed plant functional diversity to be directly related to increased nutrient availability in the soil solution (i.e., soil water sulfate, potassium and calcium) as well as to increased soil phosphate [32,36,37]. Such findings can be explained by the complementarity effects of plant diversity on nutrients, thus, reinforcing the need for restoring functional diversity in degraded grasslands. Contrarily, the structural equation models presented here indicated that plant functional diversity indirectly controlled nutrient concentrations in the soil solution via biomass production, potentially reducing nutrient availability by consumption. This pattern can be explained by the diversity-productivity relationship, in which the higher biomass production would lead to an increasing ability of plants to explore, acquire and store nutrients [38]. Plants acquire different nutrient portions from distinct nutrient types as biomass production increases with diversity [3,39]. Therefore, it is less likely that plants from functionally diverse communities will contribute to the exhaustion of single nutrients because having higher ecological niche differentiation and occupancy, particularly, at smaller scales when competitive interactions drive species performances and ecological differences play an important role [40,41]. Furthermore, plant diversity can positively influence soil fertility not only by complementarily using soil resources but also by having a strong positive effect on litter quality due to producing an organic matter pool, which will be more chemically and biologically labile [42]. 

In turn, presence of *S. gigantea* disrupted the effect of functional diversity on plant biomass and reduced phosphate consumption by almost 50% in the invaded communities, which can lead to phosphate accumulation in invaded soils [19,20,23]. Further, the reduction in calcium availability found in invaded communities can compromise native plant biomass production in the long term. Such changes in soil water calcium and phosphate could be significant factors reinforcing *S. gigantea* invasion success. Moreover, the successful invasion of *S. gigantea* can also result from impacting soil nutrients through its effects on soil pH [18,19]. Invasive plants can strongly affect concentrations of both anion and cation in invaded soils [12]. Indeed, concentrations of sulfate, potassium, magnesium and calcium in invaded soils were indirectly negatively affected by *S. gigantea* via the effects of aboveground biomass on soil pH, corroborating the expected effects of invasive plants on nutrient cation forms [12]. 

Our findings seem to contradict the idea that invasive plants increase soil fertility in invaded sites [19,20,23,34]. On one hand, such pattern can be partially explained by an increased consumption effect resulting from the addition of *S. gigantea* plants in our artificially invaded communities. This indicates that *Solidago* plants might adopt a strategy of high nutrient consumption during early stages of invasion to outcompete native plants in terms of biomass production. On the other hand, if considering a long-term perspective, *S. gigantea* plants might increase soil nutrient levels due to the recycling of organic matter with high nutrient content. Although not assessed here, *S. gigantea* is known to increase mineralization rates and to have a higher nutrient uptake rate as well as nutrient-use efficiency [19,20]. Such effect caused by *S. gigantea* might corroborate the argument for a strong depletion of nutrients in early stages with a high nutrient turnover in the long term. 

Our work identified initial impacts of plant invasions on grassland communities. Nevertheless, short-term effects of invasive plants on ecosystems can be less pronounced than long-term effects [13]. Thus, our results need to be complemented by long-term assessments to fully understand how invasive plants influence ecosystem functioning in restored grasslands. 

## 4. Material and Methods

### 4.1. Plant Trait Selection and Functional Groups

Functional group classification was performed according to Yannelli et al. (2017) [37] and resulted in three different functional groups (Figure S2). We used trait information for 54 native grassland species (Table S1). We selected eight traits known as good proxies for species dispersal, establishment success, growth, persistence and competitive ability [33,43,44], i.e., specific leaf area (SLA; g cm^−2^), leaf dry matter (mg), life form, shoot morphology, root morphology, canopy height at maturity (m), seed mass (g) and longevity. While SLA, canopy height at maturity and seed mass can be correlated with competition [45] and invasiveness [46]; longevity (i.e., annual or perennial plants), life form and morphological aspects can be related to temporal and spatial niche overlap or differentiation [36] as well as to temporal resource acquisition [47]. Leaf dry matter, in turn, can account for rates of nutrient mineralization [48,49]. Functional trait information was obtained from BiolFlor [50,51] and LEDA databases [52]. 

Finally, for making sure that the functional composition of the mesocosms (functional group, FG 1–3) resulted in plant communities with increasing levels of functional diversity, we calculated functional dispersion and redundancy indexes [28,53] and correlated such indexes to functional group classification using a linear regression model. Details on functional groups clustering and on the calculation of functional diversity indexes can be found in the Supplementary material (Supplementary File 2, Table S1; Figure S2, Table S2; Figure S3).

### 4.2. Experimental Design

The mesocosm experiment had a randomized block design, started in late November 2013 and ran over 16 weeks within the Centre of Greenhouses and Laboratories Dürnast, Technical University of Munich (48° 24′ N, 11° 41′ E). The experiment was conducted in a heated greenhouse using plastic trays with 48.2 × 33 × 6.2 cm^3^, i.e., approximately 0.16 m^2^ of area and 0.0098 m^3^ of volume. Trays were filled with circa 9.8 l of potting soil consisting in a mixture of peat, quartz sand and clay powder (2:1:1, Floragard Vertriebs GmbH in Oldenburg, Germany), arranged within five blocks. Artificial light was provided during 16 h per day (4–15 ± 0.5 lux); daily temperatures were 16–21 °C. Plants were watered every two days using tap water with the following anion content, i.e., 38.7 µg L^−1^ chloride, 0.0001 µg L^−1^ nitrate, 15.9 µg L^−1^ phosphate and 28.9 µg L^−1^ sulfate.

We used a factorial design with six treatment combinations: communities with three levels of functional diversity (1, 2 or 3 functional groups), with and without the invasive alien species *S. gigantea* (+S, −S). A control treatment (bare soil) was also established to assess soil nutrient conditions without plant influence. Functional group composition was randomly selected for each replicate. The grassland communities were designed by randomly selecting nine species from a regional pool of native plants according to the number of functional groups in each treatment. If two species from the same genus were selected by chance, one was replaced by another species from the same group (see Supplementary File 2 for community composition). 

The invasive perennial plant, *S. gigantea* (Asteraceae), native to North America, was selected as a model species because it is a widespread and successful invader in Central Europe [19,29]. The species invades a broad range of habitats in Europe, from drylands to wetlands and from nutrient-poor to nutrient-rich sites [26,35]. Seed sowing was carried out at a density of 3 g m^−2^ for the native target community and 1 g m^−2^ for *S. gigantea*. Densities for the native species correspond to common practice in grassland restoration for central Europe [54]. All treatment combinations were replicated five times, with a total of 40 trays.

### 4.3. Measurements

Treatment effects were evaluated by taking samples of soil and soil water during the experimental period. Five soil samples (0–5 cm depth) were collected at each mesocosm combination 5 weeks after sowing and mixed up to a single bulk. One sample was taken at each corner and one at the center of the mesocosms [29]. All soil samples were kept at −4 °C for 3 weeks when they were dried at 75 °C for 48 h, before preparation for the analysis. Subsamples (5 g) from the collected soil were taken and diluted in 100 mL of distilled water (1:20 dilution) for aqueous extraction of the soil nutrients to be analyzed. From this, 10 mL of the solution was centrifuged during a 10-min period. Furthermore, 8 mL from the centrifuged soil solutions was pipetted into plastic polyvials and frozen again for later analysis.

Soil water samples were collected 8 weeks after sowing using suction cups (2.5 mm diameter mini plastic suction cups—ecoTech GmbH). Soil water was collected during a 70-h period with a vacuum pump. For soil and soil water samples, the contents of ammonium, calcium, magnesium, phosphate, potassium and sulfate were determined using the Dionex ICS-1600 Ion Chromatography System (Thermo Fisher Scientific Inc., Waltham, MA, USA). Nutrient availability or accumulation resulting from the diversity and/or invasive species effects in each mesocosm was determined by subtracting the concentrations in the soil and soil water samples of the different communities from the concentrations obtained in the control (bare soil) mesocosms in the same period. Finally, soil reaction was measured with a pH meter (pH 196—WTW), to identify the potential for ion exchange between soil and plants.

Native and *S. gigantea* plant emergence was evaluated one week after sowing. A rectangular grid (30.5 × 46 cm^2^) divided by three columns and five rows (15 plots) was placed above the trays and five plots of this grid were randomly selected. The number of emerged plants for each selected plot was counted. Competition effects among plants were evaluated 16 weeks after seed sowing by collecting aboveground biomass for both native and invasive species. This was done by harvesting all aboveground plants (from 1 cm above soil surface), then placing native and invasive species in different paper bags. All samples were dried at 65 °C for 48 h and weighted immediately after.

### 4.4. Statistical Analysis

A linear mixed-effect model with block and species composition as random effects and the likelihood ratio test (LRT) implemented by the package lme4 [55] was applied to test for effects of functional dispersion (Fdis), invasive species presence, and their interaction on native plant emergence and aboveground biomass as well as on soil and soil water nutrients. The same model was applied to check for the effects of *S. gigantea* on total aboveground biomass of plant communities. Total plant emergence was used as covariate in the biomass models to control for possible density effects of emerged plants. Native plant biomass and total aboveground of plant communities were log-transformed before running the models. The effect of increasing functional diversity on biotic resistance was tested against *S. gigantea* aboveground biomass (log-transformed values) and emergence (Supplementary File 1; Figure S1) by applying a linear mixed-effects model with block and species composition as random terms using the package nlme in R [56].

Finally, to test if effects of functional diversity and *S. gigantea* on resource capture of the plant communities were direct or indirect, we calculated a structural equation model (SEM) using the package piecewiseSEM in R [31]. We evaluated if diversity effects would cascade through plant biomass and soil pH before they affect soil and soil water nutrients. This analysis allows statistically testing the causal relationships among variables by multilevel path models [57,58]. SEM models were implemented using mixed-effects structure of analysis (LME) to incorporate block as a random effect [31]. Statistical analyses were performed using R Statistical Computing version 3.3.1 [59].

## 5. Conclusions

Our results from a mesocosm experiment indicate that plant functional diversity partially controls soil fertility in grassland communities. However, functional diversity could not prevent impacts of the invasive *S. gigantea* on the plant community. The invasive species can compromise long-term functioning of grasslands since it interferes with community biomass, nutrient availability and stocks in the soil of invaded systems. Further experiments should test different plant traits and species to overcome the ecological strategies of *S. gigantea,* thus reducing its invasion success.

## Supplementary Materials

The following are available online at https://www.mdpi.com/2223-7747/9/1/53/s1. Figure S1: Functional classification for a set of 54 grassland plant species by trait similarity into three functional groups. Figure S2: Regression analysis for the functional dispersion and functional redundancy indexes [28,52]. The figures show the functional dispersion index (**a**) and the functional redundancy index (**b**) according to the number of functional groups composing the experimental communities and the correlation between functional dispersion and functional redundancy (**c**). Figure S3: Effects of functional dispersion on *Solidago* plants emergence and aboveground biomass. Figure (**a**) shows *S. gigantea* emergence, while (**b**) represents results for *S. gigantea* biomass varying according to the functional dispersion index. Aboveground biomass was collected at the end of the experiment (16 weeks), while emergence was evaluated during the second week of the experimental period for each one of the communities, invaded or not. For the dependent variables, F-values are presented in the text (*** *p* ≤ 0.001; ** *p* ≤ 0.01; * *p* ≤ 0.05; *p* ≤ 0.10; ns *p* > 0.10). Table S1: Functional trait characteristics for each functional group. Values of quantitative functional traits represent mean (±SD). Table S2: Functional trait characteristics of the native species occurring in the mesocosm communities used for the calculation of the functional dispersion index. Zero (0) and 1 values represent the presence/absence of categorical functional traits. Quantitative functional traits are represented by mean values for each species. Species relative abundances were determined by multiplying the 3 g m^−2^ of native species sown (i.e., 0.48 g of seeds which means, approximately, 0.054 g for each one of the native species present) by its mean seed mass to obtain the number of seeds per native species. Mean seed mass information (given in mg) was obtained from BiolFlor database [48,49]. For the species *Buphthalmum salicifolium* L. we used the thousand seed mass obtained from Rieger-Hofmann GmbH (Catalogue 2016/2017) to calculate the mean seed mass.

## References

[1] Balvanera P., Siddique I., Dee L., Paquette A., Isbell F., Gonzalez A., Byrnes J., O’Connor M.I., Hungate B.A., Griffin J.N. (2014). Linking Biodiversity and Ecosystem Services: Current Uncertainties and The Necessary Next Steps. Bioscience.

[2] Bello F.D., Lavorel S., Díaz S., Harrington R., Cornelissen J.H.C., Bardgett R.D., Berg M.P., Cipriotti P., Feld C.K., Hering D. (2010). Towards an Assessment of Multiple Ecosystem Processes and Services via Functional Traits. Biodivers. Conserv..

[3] Conti G., Díaz S. (2013). Plant Functional Diversity and Carbon Storage—An Empirical Test in Semi-Arid Forest Ecosystems. J. Ecol..

[4] Fornara D.A., Tilman D. (2008). Plant Functional Composition Influences Rates of Soil Carbon and Nitrogen Accumulation. J. Ecol..

[5] Roscher C., Schumacher J., Gubsch M., Lipowsky A., Weigelt A., Buchmann N., Schmid B., Schulze E.-D. (2012). Using Plant Functional Traits to Explain Diversity–Productivity Relationships. PLoS ONE.

[6] Cadotte M.W. (2017). Functional Traits Explain Ecosystem Function Through Opposing Mechanisms. Ecol. Lett..

[7] Fargione J.E., Tilman D. (2005). Diversity Decreases Invasion via both Sampling and Complementarity Effects. Ecol. Lett..

[8] Tilman D., Isbell F., Cowles J.M. (2014). Biodiversity and Ecosystem Functioning. Annu. Rev. Ecol. Evol. Syst..

[9] Craine J.M., Tilman D., Wedin D., Reich P., Tjoelker M., Knops J. (2002). Functional Traits, Productivity and Effects on Nitrogen Cycling of 33 Grassland Species. Funct. Ecol..

[10] Bardgett R.D., Mommer L., Vries F.T.D. (2014). Going Underground: Root Traits as Drivers of Ecosystem Processes. Trends Ecol. Evol..

[11] Byun C., Blois S.D., Brisson J. (2015). Interactions between Abiotic Constraint, Propagule Pressure, and Biotic Resistance Regulate Plant Invasion. Oecologia.

[12] Ehrenfeld J.G. (2010). Ecosystem Consequences of Biological Invasions. Annu. Rev. Ecol. Evol. Syst..

[13] Elgersma K.J., Ehrenfeld J.G., Yu S., Vor T. (2011). Legacy Effects Overwhelm the Short-Term Effects of Exotic Plant Invasion and Restoration on Soil Microbial Community Structure, Enzyme Activities, and Nitrogen Cycling. Oecologia.

[14] Levine J.M., Adler P.B., Yelenik S.G. (2004). A Meta-Analysis of Biotic Resistance to Exotic Plant Invasions. Ecol. Lett..

[15] Byun C., Blois S.D., Brisson J. (2013). Plant Functional Group Identity and Diversity Determine Biotic Resistance to Invasion by An Exotic Grass. J. Ecol..

[16] Vilà M., Espinar J.L., Hejda M., Hulme P.E., Jarošík V., Maron J.L., Pergl J., Schaffner U., Sun Y., Pyšek P. (2011). Ecological Impacts of Invasive Alien Plants: A Meta-Analysis of their Effects on Species, Communities and Ecosystems. Ecol. Lett..

[17] Vilà M., Weiner J. (2004). Are Invasive Plant Species better Competitors than Native Plant Species—Evidence from Pair-Wise Experiments. Oikos.

[18] Weidenhamer J.D., Callaway R.M. (2010). Direct and Indirect Effects of Invasive Plants on Soil Chemistry and Ecosystem Function. J. Chem. Ecol..

[19] Herr C., Chapuis-Lardy L., Dassonville N., Vanderhoeven S., Meerts P. (2007). Seasonal Effect of the Exotic Invasive Plant *Solidago gigantea* on Soil pH and P Fractions. J. Plant. Nutr. Soil Sci..

[20] Chapuis-Lardy L., Vanderhoeven S., Dassonville N., Koutika L.-S., Meerts P. (2006). Effect of the Exotic Invasive Plant *Solidago gigantea* on Soil Phosphorus Status. Biol. Fertil. Soils.

[21] Ehrenfeld J.G., Kourtev P., Huang W. (2001). Changes in Soil Functions Following Invasions of Exotic Understory Plants in Deciduous Forests. Ecol. Appl..

[22] Jakobs G., Weber E., Edwards P.J. (2004). Introduced Plants of the Invasive *Solidago gigantea* (Asteraceae) are Larger and Grow Denser than Conspecifics in the Native Range. Divers. Distrib..

[23] Dassonville N., Vanderhoeven S., Vanparys V., Hayez M., Gruber W., Meerts P. (2008). Impacts of Alien Invasive Plants on Soil Nutrients are Correlated with Initial Site Conditions in NW Europe. Oecologia.

[24] Hinsinger P. (2001). Bioavailability of Soil Inorganic P in the Rhizosphere as Affected by Root-Induced Chemical Changes: A Review. Plant Soil.

[25] Diekmann M., Jandt U., Alard D., Bleeker A., Corcket E., Gowing D.J.G., Stevens C.J., Duprè C. (2014). Long-Term Changes in Calcareous Grassland Vegetation in North-Western Germany—No Decline in Species Richness, but a Shift in Species Composition. Biol. Conserv..

[26] Scharfy D., Güsewell S., Gessner M.O., Venterink H.O. (2010). Invasion of *Solidago gigantea* in Contrasting Experimental Plant Communities: Effects on Soil Microbes, Nutrients and Plant-Soil Feedbacks. J. Ecol..

[27] Staab K., Yannelli F.A., Lang M., Kollmann J. (2015). Bioengineering Effectiveness of Seed Mixtures for Road Verges: Functional Composition as a Predictor of Grassland Diversity and Invasion Resistance. Ecol. Eng..

[28] Laliberté E., Legendre P. (2010). A Distance-Based Framework for Measuring Functional Diversity from Multiple Traits. Ecology.

[29] Güsewell S., Jakobs G., Weber E. (2006). Native and Introduced Populations of *Solidago gigantea* Differ in Shoot Production but not in Leaf Traits or Litter Decomposition. Funct. Ecol..

[30] Ehrenfeld J. (2003). Effects of Exotic Plant Invasions on Soil Nutrient Cycling Processes. Ecosystems.

[31] Lefcheck J.S. (2016). PiecewiseSEM: Piecewise Structural Equation Modelling in R for Ecology, Evolution, and Systematics. Methods Ecol. Evol..

[32] Han Y., Buckley Y.M., Firn J. (2012). An Invasive Grass Shows Colonization Advantages over Native Grasses Under Conditions of Low Resource Availability. Plant Ecol..

[33] Funk J.L., Cleland E.E., Suding K.N., Zavaleta E.S. (2008). Restoration through Reassembly: Plant Traits and Invasion Resistance. Trends Ecol. Evol..

[34] Sitzia T., Campagnaro T., Kotze D.J., Nardi S., Ertani A. (2018). The Invasion of Abandoned Fields by a Major Alien Tree Filters Understory Plant Traits in Novel Forest Ecosystems. Sci. Rep..

[35] Vanderhoeven S., Dassonville N., Chapuis-Lardy L., Hayez M., Meerts P. (2006). Impact of the Invasive Alien Plant *Solidago gigantean* on Primary Productivity, Plant Nutrient Content and Soil Mineral Nutrient Concentrations. Plant Soil.

[36] Clark D.L., Wilson M., Roberts R., Dunwiddie P.W., Stanley A., Kaye T.N. (2012). Plant Traits—A Tool for Restoration?. Appl. Veg. Sci..

[37] Yannelli F.A., Koch C., Jeschke J.M., Kollmann J. (2017). Limiting Similarity and Darwin’s Naturalization Hypothesis: Understanding the Drivers of Biotic Resistance Against Invasive Plant Species. Oecologia.

[38] Fornara D.A., Tilman D. (2009). Ecological Mechanisms Associated with the Positive Diversity-Productivity Relationship in an N-Limited Grassland. Ecology.

[39] Craven D., Isbell F., Manning P., Connolly J., Bruelheide H., Ebeling A., Roscher C., Van Ruijven J., Weigelt A., Wilsey B. (2016). Plant Diversity Effects on Grassland Productivity are Robust to both Nutrient Enrichment and Drought. Philos. Trans. R. Soc. Lond. B.

[40] Cadotte M.W., Carscadden K., Mirotchnick N. (2011). Beyond Species: Functional Diversity and the Maintenance of Ecological Processes and Services. J. Appl. Ecol..

[41] Laughlin D.C. (2014). Applying Trait-Based Models to Achieve Functional Targets for Theory-Driven Ecological Restoration. Ecol. Lett..

[42] Sitzia T., Pizzeghello D., Dainese M., Ertani A., Carletti P., Semenzato P., Nardi S., Cattaneo D. (2014). Topsoil Organic Matter Properties in Contrasted Hedgerow Vegetation Types. Plant Soil.

[43] Cornelissen J.H.C., Lavorel S., Garnier E., Díaz S., Buchmann N., Gurvich D.E., Reich P.B., Steege H.T., Morgan H.D., Van Der Heijden M.G.A. (2003). A Handbook of Protocols for Standardised and Easy Measurement of Plant Functional Traits Worldwide. Aust. J. Bot..

[44] Westoby M., Falster D.S., Moles A.T., Vesk P.A., Wright I.J. (2002). Plant Ecological Strategies: Some Leading Dimensions of Variation Between Species. Annu. Rev. Ecol. Syst..

[45] Garnier E., Navas M.-L. (2012). A Trait-Based Approach to Comparative Functional Plant Ecology: Concepts, Methods and Applications for Agroecology. A Review. Agron. Sustain. Dev..

[46] Hamilton M.A., Murray B.R., Cadotte M.W., Hose G.C., Baker A.C., Harris C.J., Licari D. (2005). Life-History Correlates of Plant Invasiveness at Regional and Continental Scales. Ecol. Lett..

[47] Ebeling A., Pompe S., Baade J., Eisenhauer N., Hillebrand H., Proulx R., Roscher C., Schmid B., Wirth C., Weisser W.W. (2014). A Trait-Based Experimental Approach to Understand the Mechanisms Underlying Biodiversity–Ecosystem Functioning Relationships. Basic Appl. Ecol..

[48] Cornwell W.K., Cornelissen J.H.C., Amatangelo K., Dorrepaal E., Eviner V.T., Godoy O., Hobbie S.E., Hoorens B., Kurokawa H., Pérez-Harguindeguy N. (2008). Plant Species Traits are the Predominant Control on Litter Decomposition Rates within Biomes Worldwide. Ecol. Lett..

[49] Pérez-Harguindeguy N., Díaz S., Garnier E., Lavorel S., Poorter H., Jaureguiberry P., Bret-Harte M.S., Cornwell W.K., Craine J.M., Gurvich D.E. (2013). New Handbook for Standardised Measurement of Plant Functional Traits Worldwide. Aust. J. Bot..

[50] Klotz S. (2002). Biolflor—Eine Datenbank Mit Biologisch-Ökologischen Merkmalen Zur Flora Von Deutschland.

[51] Kühn I., Durka W., Klotz S. (2004). Biolflor—A New Plant-Trait Database as a Tool for Plant Invasion Ecology. Divers. Distrib..

[52] Kleyer M., Bekker R.M., Knevel I.C., Bakker J.P., Thompson K., Sonnenschein M., Poschlod P., Van Groenendael J.M., Klimeš L., Klimešová J. (2008). The Leda Traitbase. A Database of Life-History Traits of The Northwest European Flora. J. Ecol..

[53] Ricotta C., De Bello F., Moretti M., Caccianiga M., Cerabolini B.E.L., Pavoine S. (2016). Measuring the Functional Redundancy of Biological Communities: A Quantitative Guide. Methods Ecol. Evol..

[54] Kiehl K., Kirmer A., Donath T.W., Rasran L., Hölzel N. (2010). Species Introduction in Restoration Projects—Evaluation of Different Techniques for the Establishment of Semi-Natural Grasslands in Central and Northwestern Europe. Basic Appl. Ecol..

[55] Bates D., Mächler M., Bolker B., Walker S. (2015). Fitting Linear Mixed-Effects Models Using Lme4. J. Stat. Soft..

[56] Pinheiro J., Bates D., Debroy S., Sarkar D., R Core Team (2016). Nlme: Linear and Nonlinear Mixed Effects Models. Http://Cran.R-Project.Org/Package=Nlme.

[57] Oliveira B.F., Machac A., Costa G.C., Brooks T.M., Davidson A.D., Rondinini C., Graham C.H. (2016). Species and Functional Diversity Accumulate Differently in Mammals. Glob. Ecol. Biogeogr..

[58] Shipley B. (2009). Confirmatory Path Analysis in a Generalized Multilevel Context. Ecology.

[59] R Development Core Team (2015). R: A Language and Environment for Statistical Computing.

