# Supplementary material for: Functional Diversity and Invasive Species Influence Soil Fertility in Experimental Grasslands
Source: Plants (Basel). 2020 Jan 1;9(1):53. doi: 10.3390/plants9010053 (PMC7020219; doi:10.3390/plants9010053)
Supplement: Supplementary file 1 [file plants-09-00053-s001.pdf]

Supporting information to Teixeira et al., Functional diversity and invasive species influence soil fertility in experimental grasslands

Supporting Information may be also found in the online version of this article:

List of figures:

Figure S1: Effects of functional dispersion on *Solidago* plants emergence and aboveground biomass. Figure **(a)** shows *S. gigantea* emergence, while **(b)** represents results for *S. gigantea* biomass varying according to the functional dispersion index. Aboveground biomass was collected at the end of the experiment (16 weeks), while emergence was evaluated during the second week of the experimental period for each one of the communities, invaded or not. For the dependent variables, F-values are presented in the text (*** $p \leq 0.001$; ** $p \leq 0.01$; * $p \leq 0.05$; **.** $p \leq 0.10$; ns $p > 0.10$).

Figure S2: Functional classification for a set of 54 grassland plant species by trait similarity into three functional groups.

Figure S3: Regression analysis for the functional dispersion and functional redundancy indexes [1,2]. The figures show the functional dispersion index (**a**) and the functional redundancy index (**b**) according to the number of functional groups composing the experimental communities and the correlation between functional dispersion and functional redundancy (**c**).

List of tables:

Table S1: Functional trait characteristics for each functional group. Values of quantitative functional traits represent mean (±SD).

Table S2: Functional trait characteristics of the native species occurring in the mesocosm communities used for the calculation of the functional dispersion index. Zero (0) and 1 values represent the presence/absence of categorical functional traits. Quantitative functional traits are represented by mean values for each species. Species relative abundances were determined by multiplying the 3 g m^{-2} of native species sown (i.e. 0.48 g of seeds which means, approximately, 0.054 g for each one of the native species present) by its mean seed mass to obtain the number of seeds per native species. Mean seed mass information (given in mg) was obtained from BiolFlor database [3,4]. For the species *Buphthalmum salicifolium* L. we used the thousand seed mass obtained from Rieger-Hofmann GmbH (Catalogue 2016/2017) to calculate the mean seed mass.

Supplementary File 1: Functional diversity and biotic resistance

S1.1. Functional dispersion effects on Solidago *plants emergence and aboveground biomass*

Although no statistical differences were found ($F = 0.3$, $df = 8$, $p > 0.05$ for *Solidago* biomass; and $F = 0.4$, $df = 8$, $p > 0.05$ for emergence of *Solidago* plants), emergence of *Solidago gigantea* showed a slightly negative tendency when in communities with higher functional dispersion. However, such effects were not strong enough to affect invasive plants emergence rates by competition (Fig. S1).

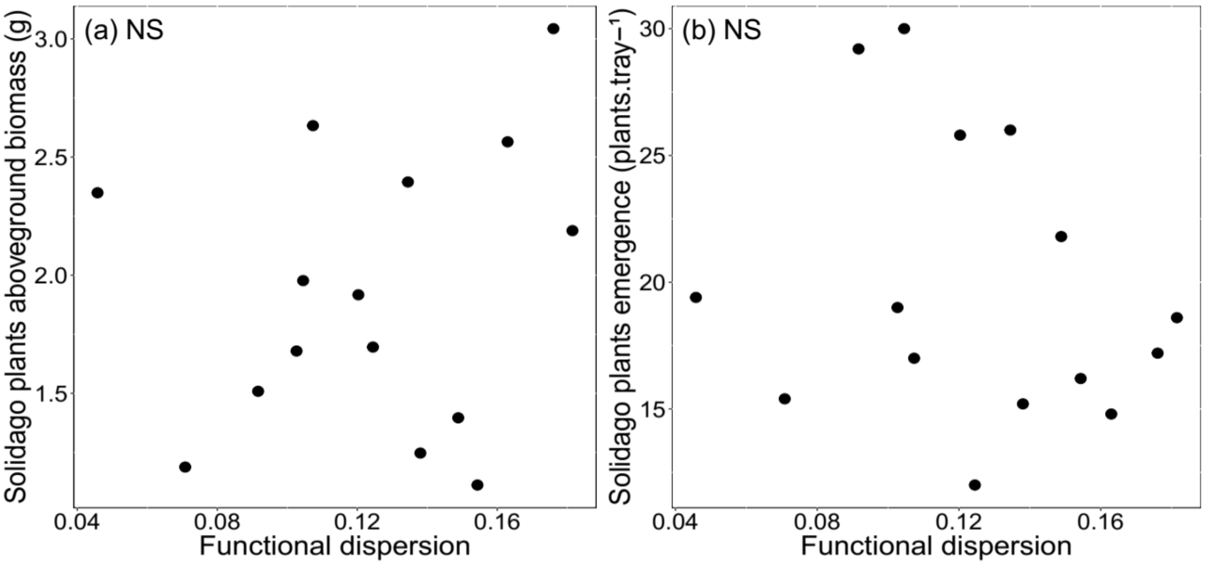

Figure S1: Effects of functional dispersion on *Solidago* plants emergence and aboveground biomass. Figure **(a)** shows *S. gigantea* biomass, while **(b)** represents results for *S. gigantea* emergence varying according to the functional dispersion index. Aboveground biomass was collected at the end of the experiment (16 weeks), while emergence was evaluated during the second week of the experimental period for each one of the communities, invaded or not. For

the dependent variables, F-values are presented in the text (*** $p \leq 0.001$; ** $p \leq 0.01$; * $p \leq 0.05$; **.** $p \leq 0.10$; NS $p > 0.10$).

Supplementary File 2: Material and methods extended

S2.1. Plant species and functional traits selection

Solidago gigantea (Asteraceae) was used as a model species, given that is known to be problematic in disturbed areas such as roadsides and newly re-vegetated areas [5]. Seeds from *S. gigantea* were collected from seven (clonal) stands along River Isar near Freising, southern Germany (48°24'N, 11°41'E). Before the experiment, the seeds were tested for viability under controlled conditions consisting in an 8 h night at 12 °C and a 16 h day at 20 °C and resulting in a germination success of 73.6 ± 4.5%. The experimental pool of native species was based on a set of 54 native grassland species occurring with a frequency ≥10% in a dataset comprising more than 100 surveys of calcareous grasslands in the agricultural landscape north of Munich, Germany [6]. The native seed material was obtained from the local seed producer Johann Krimmer (Pulling, Germany).

Functional group classification was performed using trait information for the set of 54 native grassland species (Table S1, Fig. S2). For doing so, eight traits were found to be suitable proxies for species dispersal, establishment success, growth, persistence and competitive ability [7–9], i.e. specific leaf area (SLA), leaf dry matter, life form, shoot morphology, morphology of vegetative organs, canopy height at maturity, seed mass and longevity (Table S1, Fig. S2). This was done, because SLA, canopy height at maturity and seed mass are correlated with invasiveness [10], and several traits are correlated to

competition among plant species, i.e. SLA, seed mass and canopy height at maturity. We collected the functional traits information from the BiolFlor [3,4] and LEDA databases [11].

S2.2. Functional groups clustering

Statistical analyses for the functional grouping were performed using Infostat software [12]. Previous to clustering analysis, all non-numerical functional traits were transformed into dummy variables (binary values). Subsequently, the trait information was converted to continuous values using a principal coordinate analysis using Jaccard's distance measure and saving the first five principal coordinates [13]. Based on the collected data for all functional traits, a cluster analysis was conducted using Gower's similarity coefficient among species and Ward as the linkage method [14,15]. The classification of all species led to three statistically different functional groups (Fig. S2).

To prove that clusters were significantly different, a multivariate analysis of variances was performed with the same variables. Null hypothesis of equal vector means was rejected ($F = 13.6$, $p < 0.0001$) and mean vector comparisons showed significant differences among the clusters (Table S1). Finally, we calculated functional dispersion and redundancy indexes [1,2] to check for the functional dissimilarity in our experimental communities (Appendix S2, Fig. S3).

Table S1: Functional trait characteristics for each functional group. Values of quantitative functional traits represent mean (±SD).

Functional traits	Functional group FG 1	FG 2	FG 3
Longevity	Perennial	<u>Perennial</u> + biannual + annual	Perennial
Life form	<u>Hemicryptophytes</u>, geophytes, chamaephytes	<u>Hemicryptophytes</u>	<u>Hemicryptophytes</u>, chamaephytes
Shoot morphology	<u>Hemi-rosette</u>, erosulate	<u>Hemi-rosette</u>	<u>Erosulate,</u> rosette
Root morphology	<u>Runner</u>, rhizome, tuft, pleicorm	<u>Pleiocorm,</u> runner	<u>Pleiocorm</u>, rhizome, runner
Seed mass (g)	1.60 ± 1.72	2.44 ± 1.64	1.97 ± 3.27
Canopy height (m)	0.37 ± 0.18	0.33 ± 0.22	0.27 ± 0.19
SLA (mm^{-2} mg^{-1})	22.6 ± 9.2	21.5 ± 7.2	23.2 ± 6.0
Dry leaf mass ($mg\ g^{-1}$)*	268 ± 78[b]	240 ± 44[b,a]	203 ± 52[a]

Dry leaf mass was the only quantitative trait to significantly differ among communities (ANOVA test; $F = 5.34$; $p \leq 0.05$). Values with a common letter are not significantly different ($p > 0.05$).

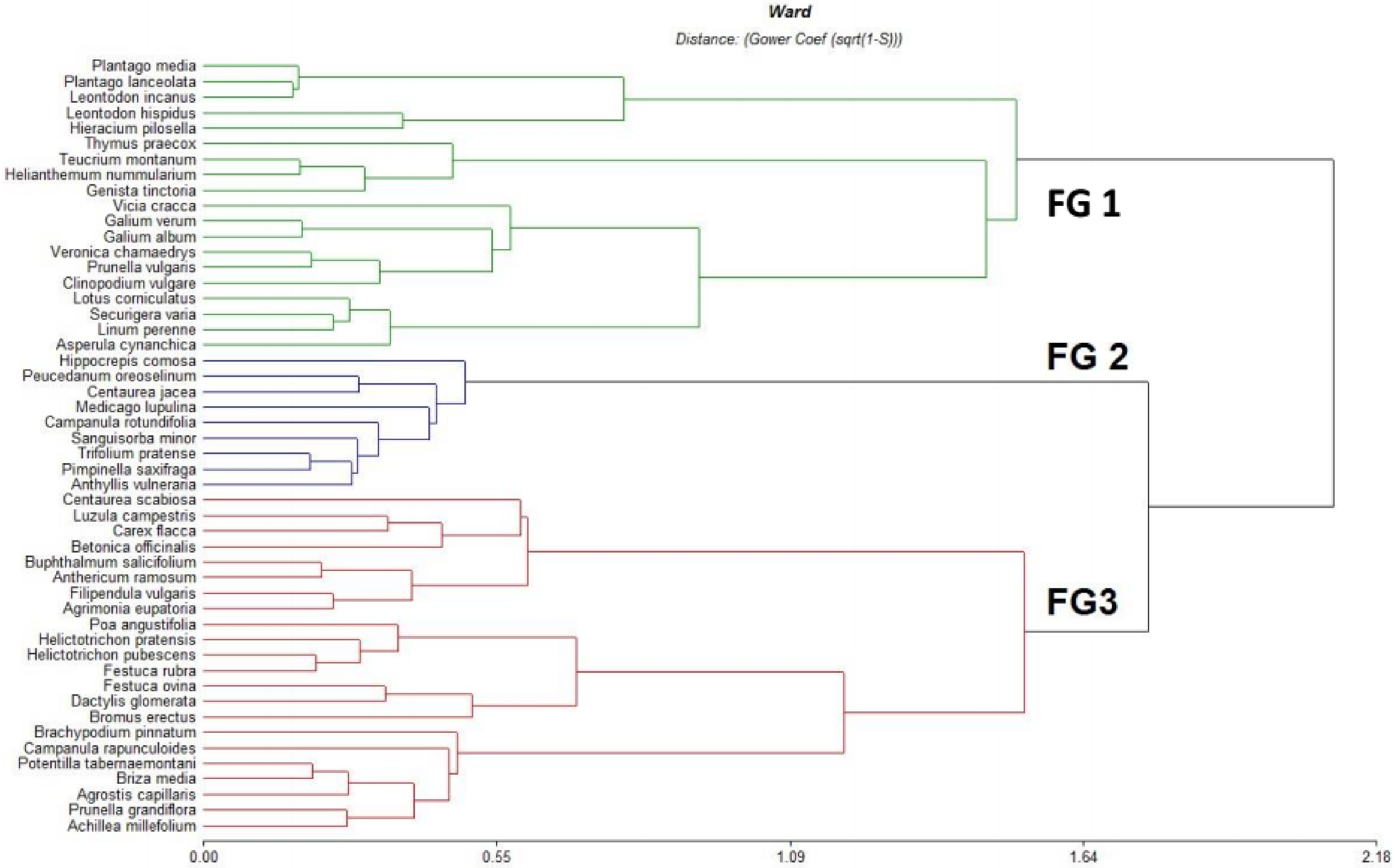

Figure S2: Functional classification for a set of 54 grassland plant species by trait similarity into three functional groups.

S2.3. Composition of the plant communities

One functional group

Replica 1 = FG1

Nr	Species	Family	FG
1	*Prunella vulgaris*	Lamiaceae	1
2	*Plantago media*	Plantaginaceae	1
3	*Helianthemum nummularium*	Cistaceae	1
4	*Genista tinctoria*	Fabaceae	1
5	*Asperula cynanchica*	Rubiaceae	1
6	*Veronica chamaedrys*	Scrophulariaceae	1
7	*Lotus corniculatus*	Fabaceae	1
8	*Hieracium pilosella*	Asteraceae	1
9	*Vicia cracca*	Fabaceae	1

Replica 2 = FG2

Nr	Species	Family	FG
1	*Anthyllis vulneraria*	Fabaceae	2
2	*Campanula rotundifolia*	Campanulaceae	2
3	*Centaurea jacea*	Asteraceae	2
4	*Hippocrepis comosa*	Fabaceae	2
5	*Medicago lupulina*	Fabaceae	2
6	*Peucedanum oreoselinum*	Apiaceae	2
7	*Pimpinella saxifraga*	Apiaceae	2
8	*Trifolium pratense*	Fabaceae	2
9	*Sanguisorba minor*	Rosaceae	2

Replica 3 = FG3

Nr	Species	Family	FG
1	*Betonica officinalis*	Lamiaceae	3
2	*Poa angustifolia*	Poaceae	3
3	*Festuca rubra*	Poaceae	3
4	*Agrimonia eupatoria*	Rosaceae	3
5	*Prunella grandiflora*	Lamiceae	3
6	*Helictotrichon pubescens*	Poaceae	3
7	*Agrostis capillaris*	Poaceae	3
8	*Achillea millefolium*	Asteraceae	3
9	*Potentilla tabernaemontani*	Rosaceae	3

Replica 4 = FG3

Nr	Species	Family	FG
1	*Helictotrichon pubescens*	Poaceae	3
2	*Dactylis glomerata*	Poaceae	3
3	*Agrostis capillaris*	Poaceae	3
4	*Anthericum ramosum*	Anthericaceae	3
5	*Potentilla tabernaemontani*	Rosaceae	3
6	*Helictotrichon pratense*	Poaceae	3
7	*Brachypodium pinnatum*	Poaceae	3
8	*Festuca rubra*	Poaceae	3
9	*Buphthalmum salicifolium*	Asteraceae	3

Replica 5 = FG 1

Nr	Species	Family	FG
1	*Teucrium montanum*	Plantaginaceae	1
2	*Helianthemum nummularium*	Cistaceae	1
3	*Galium album*	Rubiaceae	1
4	*Asperula cynanchica*	Rubiaceae	1
5	*Genista tinctoria*	Fabaceae	1
6	*Linum perenne*	Linaceae	1
7	*Vicia cracca*	Fabaceae	1
8	*Prunella vulgaris*	Lamiaceae	1
9	*Veronica chamaedrys*	Scrophulariaceae	1

Two functional groups

Replica 1 = FG 1 + FG 3

Nr	Species	Family	FG
1	*Leontodon incanus*	Asteraceae	1
2	*Achillea millefolium*	Asteraceae	3
3	*Linum perenne*	Linaceae	1
4	*Plantago media*	Plantaginaceae	1
5	*Brachypodium pinnatum*	Poaceae	3
6	*Lotus corniculatus*	Fabaceae	1
7	*Genista tinctoria*	Fabaceae	1
8	*Festuca ovina*	Poaceae	3
9	*Bromus erectus*	Poaceae	3

Replica 2 = FG 1 + FG 3

Nr	Species	Family	FG
1	*Helictotrichon pubescens*	Poaceae	3
2	*Teucrium montanum*	Plantaginaceae	1
3	*Agrostis capillaris*	Poaceae	3
4	*Veronica chamaedrys*	Scrophulariaceae	1
5	*Hieracium pilosella*	Asteraceae	1
6	*Genista tinctoria*	Fabaceae	1
7	*Prunella grandiflora*	Lamiceae	3
8	*Vicia cracca*	Fabaceae	1
9	*Brachypodium pinnatum*	Poaceae	3

Replica 3 = FG1+FG2

Nr	Species	Family	FG
1	*Veronica chamaedrys*	Scrophulariaceae	1
2	*Sanguisorba minor*	Rosaceae	2
3	*Clinopodium vulgare*	Lamiaceae	1
4	*Teucrium montanum*	Plantaginaceae	1
5	*Lotus corniculatus*	Fabaceae	1
6	*Medicago lupulina*	Fabaceae	2
7	*Anthyllis vulneraria*	Fabaceae	2
8	*Leontodon incanus*	Asteraceae	1
9	*Trifolium pratense*	Fabaceae	2

Replica 4 = FG2+FG3

Nr	Species	Family	FG
1	*Helictotrichon pratense*	Poaceae	3
2	*Anthyllis vulneraria*	Fabaceae	2
3	*Achillea millefolium*	Asteraceae	3
4	*Prunella grandiflora*	Lamiceae	3
5	*Medicago lupulina*	Fabaceae	2
6	*Sanguisorba minor*	Rosaceae	2
7	*Trifolium pratense*	Fabaceae	2
8	*Festuca ovina*	Poaceae	3
9	*Bromus erectus*	Poaceae	3

Replica 5 = FG 1 + FG 2

Nr	Species	Family	FG
1	*Sanguisorba minor*	Rosaceae	2
2	*Genista tinctoria*	Fabaceae	1
3	*Asperula cynanchica*	Rubiaceae	1
4	*Clinopodium vulgare*	Lamiaceae	1
5	*Peucedanum oreoselinum*	Apiaceae	2
6	*Linum perenne*	Linaceae	1
7	*Prunella vulgaris*	Lamiaceae	1
8	*Pimpinella saxifraga*	Apiaceae	2
9	*Anthyllis vulneraria*	Fabaceae	2

Three functional groups

Replica 1 = FG 1 + FG 2 +FG 3

Nr	Species	Family	FG
1	*Festuca rubra*	Poaceae	3
2	*Dactylis glomerata*	Poaceae	3
3	*Poa angustifolia*	Poaceae	3
4	*Hippocrepis comosa*	Fabaceae	2
5	*Campanula rotundifolia*	Campanulaceae	2
6	*Medicago lupulina*	Fabaceae	2
7	*Leontodon incanus*	Asteraceae	1
8	*Hieracium pilosella*	Asteraceae	1
9	*Teucrium montanum*	Plantaginaceae	1

Replica 2 = FG 1 + FG 2 +FG 3

Nr	Species	Family	FG
1	*Festuca rubra*	Poaceae	3
2	*Poa angustifolia*	Poaceae	3
3	*Centaurea scabiosa*	Asteraceae	3
4	*Sanguisorba minor*	Rosaceae	2
5	*Pimpinella saxifraga*	Apiaceae	2
6	*Anthyllis vulneraria*	Fabaceae	2
7	*Asperula cynanchica*	Rubiaceae	1
8	*Lotus corniculatus*	Fabaceae	1
9	*Vicia cracca*	Fabaceae	1

Replica 3 = FG 1 + FG 2 +FG 3

Nr	Species	Family	FG
1	*Centaurea scabiosa*	Asteraceae	3
2	*Dactylis glomerata*	Poaceae	3
3	*Potentilla tabernaemontani*	Rosaceae	3
4	*Medicago lupulina*	Fabaceae	2
5	*Campanula rotundifolia*	Campanulaceae	2
6	*Anthyllis vulneraria*	Fabaceae	2
7	*Galium album*	Rubiaceae	1
8	*Leontodon incanus*	Asteraceae	1
9	*Helianthemum nummularium*	Cistaceae	1

Replica 4 = FG 1 + FG 2 +FG 3

Nr	Species	Family	FG
1	*Betonica officinalis*	Lamiaceae	3
2	*Potentilla tabernaemontani*	Rosaceae	3
3	*Poa angustifolia*	Poaceae	3
4	*Trifolium pratense*	Fabaceae	2
5	*Sanguisorba minor*	Rosaceae	2
6	*Anthyllis vulneraria*	Fabaceae	2
7	*Galium album*	Fabaceae	1
8	*Teucrium montanum*	Plantaginaceae	1
9	*Leontodon incanus*	Asteraceae	1

Replica 5 = FG 1 + FG 2 +FG 3

Nr	Species	Family	FG
1	*Festuca ovina*	Poaceae	3
2	*Brachypodium pinnatum*	Poaceae	3
3	*Agrostis capillaris*	Poaceae	3
4	*Campanula rotundifolia*	Campanulaceae	2
5	*Pimpinella saxifraga*	Apiaceae	2
6	*Centaurea jacea*	Asteraceae	2
7	*Leontodon incanus*	Asteraceae	1
8	*Genista tinctoria*	Fabaceae	1
9	*Prunella vulgaris*	Lamiceae	1

S2.4. Functional dispersion and functional redundancy indexes

We calculated two indexes of functional diversity: **i.** The functional dispersion (Fdis) index using the dbFD function in the FD package in R [1], and **ii.** the functional redundancy (Fred) index using the R function called 'uniqueness' [2]. For calculating the indexes, we used plant traits obtained from the BiolFlor [3,4] and LEDA databases [11] as presented in Table S2. Both measures can be calculated including categorical and numerical traits and provide opposite insights on the how functional diversity is distributed amongst our experimental communities. As previously described, Fdis represents the mean distance in the multidimensional trait space comparing the values of individual species to the value of all species. In other words, Fdis indicates the degree in which plant species occupy the multidimensional trait space. Fdis can correct for different abundances among species, thus controlling possible effects of more abundant species. Another important aspect is that Fdis is not affected by the species richness values in different communities [1].

On the other hand, Fred represents the similarities amongst species composing a given community in terms of their functional traits and, consequently, their performances. Since ecosystem functioning under stressful conditions (e.g. invasions) would require the occurrence of species with similar performances, we can expect Fred to be an important component of diversity ensuring ecosystem functions over time [2]. However, despite high values of Fred can indicate that ecosystem functioning would be less affected by species loss, it can also occur when there is a weak relationship between species richness and functions [16].

Afterwards, we correlated these functional diversity indexes to the number of functional groups resulting from the cluster analysis based on plant traits using a linear model. We also correlated (using the same type of linear model) the Fdis and Fred indexes to check if they were positively or negatively correlated (Fig. S3). The number of functional groups was log-transformed prior to the analysis to fulfill the linear model assumptions. This analysis showed that both functional diversity indexes were significantly correlated to the levels of functional diversity determined by the cluster analysis using plant traits (Fig. S3). Fdis significantly increased with the number of functional groups composing the plant communities ($R^2 = 0.41$, $p \leq 0.01$, Fig. S3a), while the Fred index decreased with the number of functional groups in our experimental communities ($R^2 = 0.45$, $p \leq 0.01$, Fig. S3b). Finally, a negative correlation was found between the indexes, indicating that both measures represent opposite aspects of functional diversity and that one cannot maximize them at the same time when trying to restore a given plant community ($R^2 = 0.99$, $p \leq 0.001$, Fig. S3c).

Table S2: Functional traits characteristics of the native species occurring in our experimental communities used for the calculation of the functional dispersion index. Zero (0) and 1 values represent the presence/absence of categorical functional traits. Quantitative functional traits are represented by mean values for each species. Species relative abundances were determined by multiplying the 3 g m^{-2} of native species sown (i.e. 0.48 g of seeds which means, approximately, 0.054 g for each one of the native species present in our communities) by its mean seed mass to obtain the number of seeds per native species. Mean seed mass information (mg) was obtained from BiolFlor database [3,4]. For the species *Buphthalmum salicifolium* L. we used the thousand seed mass obtained from Rieger-Hofmann GmbH (Catalogue 2016/2017) to calculate the mean seed mass.

**Native grasslands species**	**seed mass**	**cannopy height**	**life long perennial**	**life long biannual**	**life long annual**	**life form hemicryptophytes**	**life form geophytes**	**life form chamaephytes**	**life form hph**	**root morphology rhizome**	**root morphology runner**	**root morphology pleiocorm**	**root morphology tuft**	**shoot morphology hemi-rosette**	**shoot morphology rosette**	**shoot morphology erosulate**	**non-legume**	**legume**	**grass**	**sla**	**dry leaf mass**
Achillea millefolium	0.13	0.3955	1	0	0	1	0	0	0	0	1	0	0	1	0	0	1	0	0	19.475	185.5
Agrimonia eupatoria	3.61	0.3	1	0	0	1	0	0	0	1	0	0	0	1	0	0	1	0	0	17.85	339.5
Agrostis capillaris	0.07	0.25	1	0	0	1	0	0	0	0	1	0	0	1	0	0	0	0	1	33.2	263
Anthericum ramosum	3.81	0.2875	1	0	0	1	0	0	0	1	0	0	0	1	0	0	1	0	0	23.51	161.45
Anthyllis vulneraria	3.31	0.2	1	0	0	1	0	0	0	0	0	1	0	1	0	0	0	1	0	18.85	170
Asperula cynanchica	0.97	0.2375	1	0	0	1	0	0	0	0	0	1	0	0	0	1	1	0	0	28.06	329.32
Betonica officinalis	1.39	0.375	1	0	0	1	0	0	0	1	0	0	0	0	0	1	1	0	0	25.45	227.705
Brachypodium pinnatum	3.38	0.6	1	0	0	1	0	0	0	0	1	0	0	1	0	0	0	0	1	27.33	397
Briza media	0.35	0.165	1	0	0	1	0	0	0	0	1	0	0	1	0	0	0	0	1	25.85	295.57
Bromus erectus	4.59	0.5	1	0	0	1	0	0	0	1	0	0	1	1	0	0	0	0	1	20.725	353
Buphthalmum salicifolium	0.46	0.35	1	0	0	1	0	0	0	1	0	0	0	1	0	0	1	0	0	25.72	181.45
Campanula rapunculoides	0.11	0.375	1	0	0	1	0	0	0	0	1	0	0	1	0	0	1	0	0	54.74	135.545
Campanula rotundifolia	0.07	0.19	1	0	0	1	0	0	0	0	0	1	0	1	0	0	1	0	0	36.7	255.5
Carex flacca	0.95	0.275	1	0	0	0	1	0	0	1	1	0	0	1	0	0	0	0	1	15	332.095
Centaurea jacea	1.53	0.85	1	0	0	1	0	0	0	0	0	1	0	1	0	0	1	0	0	15.65	219.59
Centaurea scabiosa	6.17	0.825	1	0	0	1	0	0	0	1	0	1	0	1	0	0	1	0	0	18.45	197
Clinopodium vulgare	0.45	0.425	1	0	0	1	0	0	0	0	1	0	0	0	0	1	1	0	0	23.6	258

Dactylis glomerata	0.9	0.454	1	0	0	1	0	0	0	0	0	0	1	1	0	0	0	0	1	31.75	212
Festuca ovina	0.33	0.1525	1	0	0	1	0	0	0	0	0	0	1	1	0	0	0	0	1	19.29	309.74
Festuca rubra	0.79	0.45	1	0	0	1	0	0	0	0	1	0	1	1	0	0	0	0	1	17	273
Filipendula vulgaris	0.72	0.35	1	0	0	1	0	0	0	1	0	0	0	1	0	0	1	0	0	13	295
Galium album	0.53	0.45	1	0	0	1	0	0	0	0	1	1	0	0	0	1	1	0	0	24.09	162.37
Galium verum	0.57	0.425	1	0	0	1	0	0	0	0	1	1	0	0	0	1	1	0	0	22.25	227
Genista tinctoria	3.86	0.45	1	0	0	0	0	0	1	0	0	0	0	0	0	1	0	1	0	16.17	248
Helianthemum nummularium	1.08	0.1155	1	0	0	0	0	0	1	0	0	0	0	0	0	1	1	0	0	14.5	237.5
Helictotrichon pratense	3.11	0.65	1	0	0	1	0	0	0	0	1	0	1	1	0	0	0	0	1	9.85	318
Helictotrichon pubescens	1.91	0.6	1	0	0	1	0	0	0	0	1	0	1	1	0	0	0	0	1	21.3	264
Hieracium pilosella	0.15	0.06	1	0	0	1	0	0	0	1	1	0	0	0	1	0	1	0	0	24.29	188
Hippocrepis comosa	3.9	0.14	1	0	0	1	0	0	0	0	1	1	0	1	0	0	0	1	0	15.75	235.66
Leontodon hispidus	0.99	0.235	1	0	0	1	0	0	0	1	0	0	0	0	1	0	1	0	0	22.17	143.64
Leontodon incanus	1.4	0.1	1	0	0	1	0	0	0	0	0	1	0	0	1	0	1	0	0	21.36	157.22
Linum perenne	2.18	0.45	1	0	0	1	0	0	0	0	0	1	0	0	0	1	1	0	0	25.88	200.98
Lotus corniculatus	1.46	0.2	1	0	0	1	0	0	0	0	0	1	0	0	0	1	0	1	0	29.5	158
Luzula campestris	0.74	0.27	1	0	0	1	0	0	0	1	1	0	0	1	0	0	0	0	1	24.85	245.74
Medicago lupulina	1.76	0.325	1	1	1	1	0	0	0	0	0	1	0	1	0	0	0	1	0	29.2	196
Peucedanum oreoselinum	3.83	0.5	1	0	0	1	0	0	0	0	0	1	0	1	0	0	1	0	0	16.27	259.32
Pimpinella saxifraga	1.13	0.3	1	0	0	1	0	0	0	0	0	1	0	1	0	0	1	0	0	17.12	285.5
Plantago lanceolata	1.8	0.15	1	0	0	1	0	0	0	0	0	1	0	0	1	0	1	0	0	16.82	140.605
Plantago media	0.36	0.05	1	0	0	1	0	0	0	0	0	1	0	0	1	0	1	0	0	18.7	137
Poa angustifolia	0.21	0.36	1	0	0	1	0	0	0	0	1	0	1	1	0	0	0	0	1	15.55	424.5
Potentilla tabernaemontani	0.63	0.1	1	0	0	1	0	0	0	0	1	0	0	1	0	0	1	0	0	16.75	315
Prunella grandiflora	0.78	0.1325	1	0	0	1	0	1	0	0	1	0	0	1	0	0	1	0	0	20.11	174.37
Prunella vulgaris	0.7	0.145	1	0	0	1	0	1	0	0	1	0	0	0	0	1	1	0	0	33.01	165
Sanguisorba minor	5.08	0.2	1	0	0	1	0	0	0	0	0	1	0	1	0	0	1	0	0	22	313.96
Securigera varia	5.4	0.425	1	0	0	1	0	0	0	0	0	1	0	0	0	1	0	1	0	32.9	168.33
Teucrium montanum	0.91	0.2	1	0	0	0	0	0	1	0	0	0	0	0	0	1	1	0	0	17.93	264.12
Thymus praecox	0.15	0.07	1	0	0	0	0	0	1	0	1	0	0	0	0	1	1	0	0	14.82	234
Trifolium pratense	1.36	0.275	1	0	0	1	0	0	0	0	0	1	0	1	0	0	0	1	0	21.5	225.13

Veronica chamaedrys	0.19	0.25	1	0	0	1	0	1	0	0	1	0	0	0	0	1	1	0	0	32.21	215.38
Vicia cracca	14.29	0.75	1	0	0	1	0	0	0	0	1	0	0	0	0	1	0	1	0	22.04	219.49

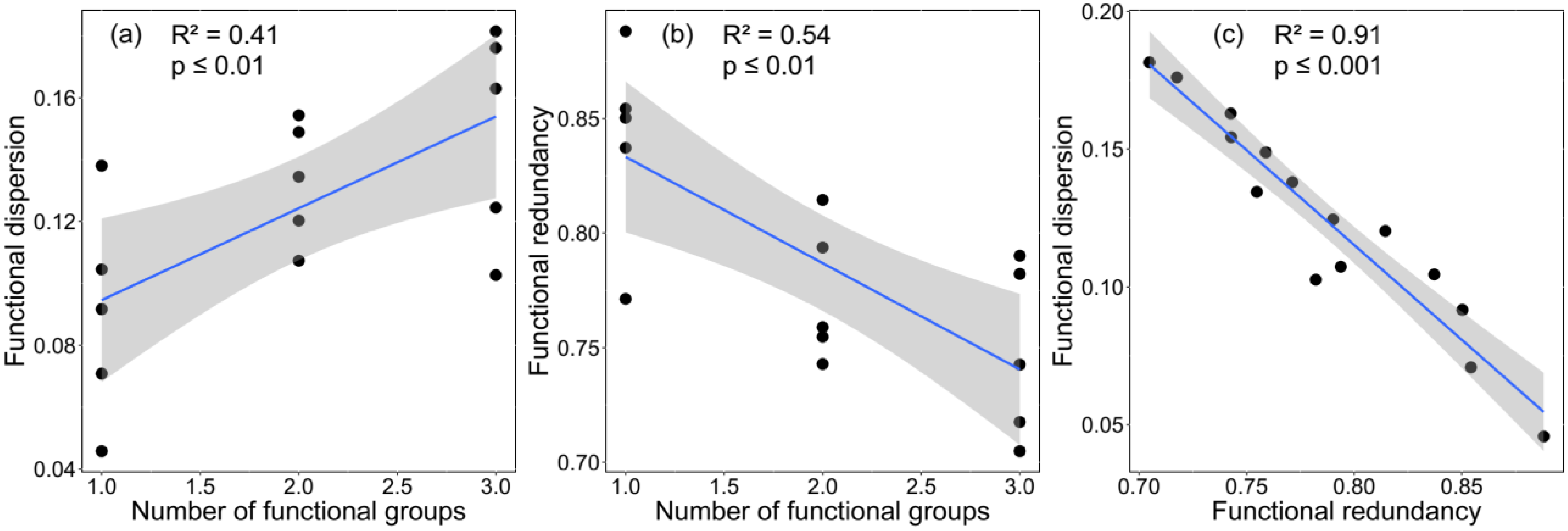

Figure S3: Regression analysis for the functional dispersion and functional redundancy indexes [1,2]. The figures show the functional dispersion index (**a**) and the functional redundancy index (**b**) according to the number of functional groups composing the experimental communities and the correlation between functional dispersion and functional redundancy (**c**).

REFERENCES

1. Laliberté, E.; Legendre, P. A distance-based framework for measuring functional diversity from multiple traits. *Ecology* **2010**, *91*, 299–305, doi:10.1890/08-2244.1.

2. Ricotta, C.; Bello, F. de; Moretti, M.; Caccianiga, M.; Cerabolini, B.E.L.; Pavoine, S. Measuring the functional redundancy of biological communities: a quantitative guide. *Methods Ecol. Evol.* **2016**, *7*, 1386–1395, doi:10.1111/2041-210X.12604.

3. Klotz, S. *BIOLFLOR - eine Datenbank mit biologisch-ökologischen Merkmalen zur Flora von Deutschland;* BfN-Schriftenvertrieb im Landwirtschaftsverl.: Münster, 2002, ISBN 378433508X.

4. Kühn, I.; Durka, W.; Klotz, S. BiolFlor - a new plant-trait database as a tool for plant invasion ecology. *Diversity and Distributions* **2004**, *10*, 363–365, doi:10.1111/j.1366-9516.2004.00106.x.

5. Kowarik, I.; Boye, P. *Biologische Invasionen. Neophyten und Neozoen in Mitteleuropa;* Ulmer: Stuttgart, 2003, ISBN 3800139243.

6. Conradi, T.; Kollmann, J. Species pools and environmental sorting control different aspects of plant diversity and functional trait composition in recovering grasslands. *J. Ecol.* **2016**, *104*, 1314–1325, doi:10.1111/1365-2745.12617.

7. Cornelissen, J.H.C.; Lavorel, S.; Garnier, E.; Díaz, S.; Buchmann, N.; Gurvich, D.E.; Reich, P.B.; Steege, H.t.; Morgan, H.D.; van der Heijden, M.G.A.; et al. A handbook of protocols for standardised and easy measurement of plant functional traits worldwide. *Aust. J. Bot.* **2003**, *51*, 335, doi:10.1071/BT02124.

8. Funk, J.L.; Cleland, E.E.; Suding, K.N.; Zavaleta, E.S. Restoration through reassembly: plant traits and invasion resistance. *Trends Ecol. Evol.* **2008**, *23*, 695–703, doi:10.1016/j.tree.2008.07.013.

9. Westoby, M.; Falster, D.S.; Moles, A.T.; Vesk, P.A.; Wright, I.J. Plant Ecological Strategies: Some Leading Dimensions of Variation Between Species. *Annu. Rev. Ecol. Syst.* **2002**, *33*, 125–159, doi:10.1146/annurev.ecolsys.33.010802.150452.

10. Hamilton, M.A.; Murray, B.R.; Cadotte, M.W.; Hose, G.C.; Baker, A.C.; Harris, C.J.; Licari, D. Life-history correlates of plant invasiveness at regional and continental scales. *Ecol. Lett.* **2005**, *8*, 1066–1074, doi:10.1111/j.1461-0248.2005.00809.x.

11. Kleyer, M.; Bekker, R.M.; Knevel, I.C.; Bakker, J.P.; Thompson, K.; Sonnenschein, M.; Poschlod, P.; van Groenendael, J.M.; Klimeš, L.; Klimešová, J.; et al. The LEDA Traitbase: a database of life-history traits of the Northwest European flora. *J. Ecol.* **2008**, *96*, 1266–1274, doi:10.1111/j.1365-2745.2008.01430.x.

12. Di-Rienzo, J., Casanoves, F., Balzarini, M., Gonzalez, L., Tablada, M., Robledo, C. (2013). InfoStat versión 2013. in F. d. C. A. InfoStat Group, Universidad Nacional de Córdoba, Argentina

13. Pla, L.; Casanoves, F.; Di Rienzo, J. *Quantifying Functional Biodiversity;* The Author(s): Dordrecht, 2012, ISBN 9789400726475.

14. Podani, J. Extending Gower's general coefficient of similarity to ordinal characters. *TAXON* **1999**, *48*, 331–340, doi:10.2307/1224438.

15. Rostagno, C.M.; Defossé, G.E.; del Valle, H.F. Postfire Vegetation Dynamics in Three Rangelands of Northeastern Patagonia, Argentina. *Rangeland Ecology & Management* **2006**, *59*, 163–170, doi:10.2111/05-020R1.1.

16. Cardinale, B.J.; Srivastava, D.S.; Duffy, J.E.; Wright, J.P.; Downing, A.L.; Sankaran, M.; Jouseau, C. Effects of biodiversity on the functioning of trophic groups and ecosystems. *Nature* **2006**, *443*, 989–992, doi:10.1038/nature05202.
